# Photocatalytic Degradation of Food and Juices Dyes via Photocatalytic Nanomaterials Synthesized through Green Synthetic Route: A Systematic Review

**DOI:** 10.3390/molecules28124600

**Published:** 2023-06-07

**Authors:** Kashif Ali Khan, Afzal Shah, Jan Nisar, Abdul Haleem, Iltaf Shah

**Affiliations:** 1 Department of Chemistry, Quaid-i-Azam University, Islamabad 45320, Pakistan; kashifali@chem.qau.edu.pk; 2 National Centre of Excellence in Physical Chemistry, University of Peshawar, Peshawar 25120, Pakistan; pashkalawati@gmail.com; 3 School of Chemistry and Chemical Engineering, Jiangsu University, Zhenjiang 212013, China; 4 Department of Chemistry, College of Science, United Arab Emirates University, Al Ain P.O. Box 15551, United Arab Emirates

**Keywords:** green synthesis, nanoparticles, dyes, photodegradation, degradation mechanism

## Abstract

The unavailability of non-poisonous and hygienic food substances is the most challenging issue of the modern era. The uncontrolled usage of toxic colorant moieties in cosmetics and food manufacturing units leads to major threats to human life. The selection of environmentally benign approaches for the removal of these toxic dyes has gained the utmost attention from researchers in recent decades. This review article’s main aim is the focus on the application of green-synthesized nanoparticles (NPs) for the photocatalytic degradation of toxic food dyes. The use of synthetic dyes in the food industry is a growing concern due to their harmful effects on human health and the environment. In recent years, photocatalytic degradation has emerged as an effective and eco-friendly method for the removal of these dyes from wastewater. This review discusses the various types of green-synthesized NPs that have been used for photocatalytic degradation (without the production of any secondary pollutant), including metal and metal oxide NPs. It also highlights the synthesis methods, characterization techniques, and photocatalytic efficiency of these NPs. Furthermore, the review explores the mechanisms involved in the photocatalytic degradation of toxic food dyes using green-synthesized NPs. Different factors that responsible for the photodegradation, are also highlighted. Advantages and disadvantages, as well as economic cost, are also discussed briefly. This review will be advantageous for the readers because it covers all aspects of dyes photodegradation. The future feature and limitations are also part of this review article. Overall, this review provides valuable insights into the potential of green-synthesized NPs as a promising alternative for the removal of toxic food dyes from wastewater.

## 1. Introduction

Dyes are widely used in various industries, including food, textile, pharmaceutics and cosmetic industries for the addition of color to products to make them more attractive for customers [1]. However, depending on their chemical composition and usage, some dyes may pose toxicity risks for aquatic organisms [2]. Water has been greatly polluted with dyes from industrial wastewater, mainly from the cosmetics, textile, paper and food industries, which companies allow to fall untreated into the natural water resources. These pollutants ultimately pose life-threatening risks to water biota, plants, and human beings, even in low concentrations, due to products produced during their degradation, particularly photodegradation [3]. In the textile industry, some azo dyes have been found to break down into toxic aromatic amines, which may cause cancer and other health problems. As a result, some countries have banned or restricted the use of certain azo dyes in textiles [4]. In the cosmetic industry, some dyes may cause skin irritation, allergic reactions, and other adverse effects, especially when used in high concentrations or on sensitive skin [5].

Dyes are mainly divided into three different categories, namely, anionic, cationic and nonionic dyes. Anionic dyes mainly possess negatively charged groups in their primary structure, such as the SO_3_^−^ group, while cationic dyes have positively charged groups, such as the protonated amine group. The third type of dyes are called nonionic due to their dissociative behavior in aqueous solutions [6]. Azo-based dyes have more stability than other dyes due to presence of the azo group, which gives them much higher stability when affected by heat, aerobic digestion, and light. However, they may also cause severe problems in human health, such as genetic mutation, allergic problems, vomiting, and cyanosis. Dyes from different industries are discharged into bodies of water where they cause damage to aquatic life. These dyes can cause suffocation in aquatic animals, such as fish, by affecting their gills. Aquatic animals’ breeding and shelter locations may also be affected. Dyes can also affect photosynthesis by blocking the passage of light through the water, causing problems for aquatic animals [7]. Because of these toxic effects, it is very necessary to remove these dyes from water bodies by some technique to save aquatic life [8].

Other types of dyes include: (a) Basic dyes such as thiazine, oxazine, azine, triaryl methyl, and cyanin derivatives. Basic dyes, known for their brilliant intense color, play important role in the textile sector [9]. The positively charged dyes attract the negatively charged anions of nylon, polyester, and acrylic fibers through electrostatic forces. (b) Acidic dyes, including methyl orange, methyl red, and erichrome black T [10]. These dyes are widely used as coloring agents for synthetic (polyamides, acrylic) fibers in acidic conditions (low pH) [11]. These dyes are also employed as food colorants [12]. (c) Reactive dyes, such as remazol brilliant red FG, reactive red 120, etc., are very reactive towards the nucleophilic sites of the fabrics. They can make strong covalent bonds with the fabric’s nucleophilic site and are applied to cotton, silk and rayon. (d) Dispersive dyes, such as dispersol, samaron, etc., are sparingly soluble and disperse in water. They attach to the fabric by hydrogen bonding and are useful for nylon, acrylic and polyester [8].

There are many man-made food and juice colorants that are commonly used in the food industry, each with their own associated potential toxicity, and the toxicity associated with each one can vary depending on the specific chemical compound [13] and the dosage [14]. Some synthetic dyes, such as Red 40, Yellow 5, and Yellow 6, have been linked to adverse health effects, such as hyperactivity in children, allergic reactions, and, in animal studies, cancer. As a result, some countries have banned or restricted the use of these dyes in food products, but many are still illegally used in those countries [15]. Some of the most common food and juice colorants that are illegally added to our food in most parts of the world, along with their uses and associated toxicity, are presented in Table 1.

## 2. Methods Used for the Degradation of Food Dyes

Various methods have been commonly employed for the degradation of toxic food dyes. The most common methods include photocatalytic degradation of dye incorporating NPs that use UV light to break down the dye molecules into smaller and less harmful compounds, most often water and carbon dioxide [29]. Another method used for degradation of dyes is biodegradation. This method involves the use of fungi and certain strains of bacteria that can break the dye molecules into smaller, harmless compounds [30]. Ozonation is one of the other most used methods for this purpose. It involves using ozone as an oxidizing agent to breakdown the dye molecules. Ozone is a strong oxidizing agent that can oxidize many organic compounds, including food dyes, into smaller, environmentally friendly compounds [31]. Chemical methods have also been employed for the degradation of dyes. This involves the use of certain chemicals, such as hydrogen peroxide or sodium hypochlorite, to break down the dye molecule [32]. Adsorption can also be used to adsorb the dye molecules from the water and food sources using certain adsorbents, such as activated carbon and hydrogels, after which the dye can be removed by physical means [33].

Of the methods discussed so far for the degradation of dyes, photocatalytic degradation using NPs possesses several advantages over other methods, including:

- High efficiency: As the NPs possess a large surface-area-to-volume ratio, they offer many active sites within a small area and can thus increase the degradation efficiency even at a smaller concentration of the dye [34].
- Selectivity: Photocatalytic degradation using NPs is a selective process that targets specific compounds and leaves the other compounds in the food matrix unharmed. This is because the photocatalysts are activated by light on a specific wavelength which can be tailored to specific dyes [35].
- Environmental friendliness: The most important advantage of using photocatalysis over the other is the environmental friendliness of this method as it would most commonly result in the production of water and carbon dioxide as the end products which are not harmful to the environment, and we can thus reduce the toxicity of the dyes to a greater extent [36].
- Versatility: photocatalytic degradation can be used to degrade many toxic organic compounds, including those that are resistant to other methods; therefore, its properties are highly versatile [37].
- Cost effectiveness: This method is relatively low cost as compared to the other as it does not involve the use of any expensive material or compound. All that is required is NPs, such as ZnO or TiO_2_, and exposure to UV light that can be obtained by exposure to the sunlight [38].

**Figure 1:**
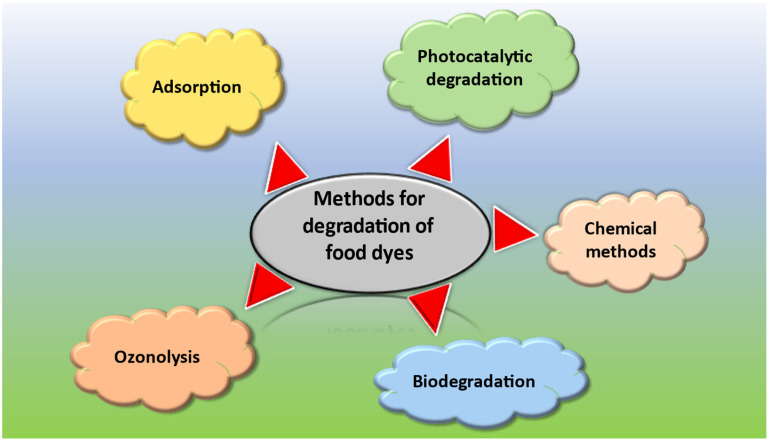
Common methods used for the degradation of food dyes.

Overall, photocatalytic degradation using NPs is a promising technique for the degradation of toxic food dyes offers several advantages over other methods. However, the efficacy of the method may depend on several factors, such as the type and concentration of the dye, nature of the food matrix, and desired level of degradation. Some of the most common methods used for the degradation of dyes are shown in Figure 1.

### 2.1. Various Approaches for Synthesis of Photocatalytic NPs

**Figure 2:**
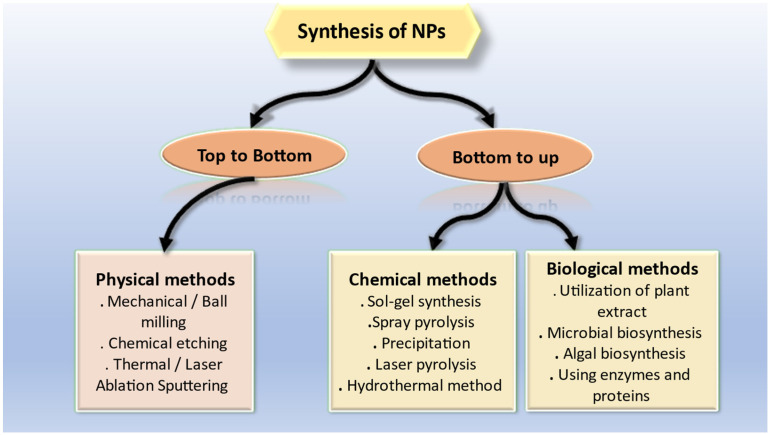
Various approaches for synthesis of NPs.

NPs are generally synthesized using two most common approaches that includes top-down and bottom-up approach. Single atoms and molecules are used in the bottom-up process to build structures. As compared to those in macro formations, the covalent forces that bind these atoms together are far greater. NPs are created first, and are then assembled to create a final structure with the desired properties. Using a top-down method, nanostructures are created by physically or chemically shrinking the size of the starting material. Some of the methods utilized in the top-down approach include cutting, carving, molding, laser ablation, electroplating, hydrothermal treatment, and nano lithography [39]. Almost any element in the periodic table can be used in nanotechnology, depending on the intended use, which can range from nanomedicine to nano sensors. Designing materials with control over size, structure, and composition at the nanoscale level results in materials with enhanced properties [39]. Physical approaches for producing NPs have some significant drawbacks, including limited productivity, high prices, and significant energy consumption to reach synthesis conditions, such as high pressure and temperature [40]. Wet chemical processes are gaining popularity as a means of overcoming the constraints of physical approaches for the creation of NPs. A common technique entails creating NPs in a liquid reaction media while specific reducing chemicals, such as hydrazine and potassium bitartrate, are present. To prevent nanoparticle aggregation, several stabilizing agents are added to the reaction mixture, such as polyvinyl pyrrolidone. Chemical processes may have certain drawbacks despite their low cost and high output, including the production of hazardous byproducts and pollution from precursor solvents [41]. Thus, the need for innovative, nontoxic, high yielding, and environmentally acceptable methods of metallic nanoparticle manufacturing is increasing. Recent years have seen a huge increase in interest in the biological approach in this area. Due to their accessibility in nature, biological resources, such as plants, microorganisms, algae, fungi, etc., could be employed to synthesize NPs [41]. Various methods that have been used so far for the synthesis of NPs are shown in Figure 2.

### 2.2. Green Synthesis and Characterization of Photocatalytic NPs

Green-synthesized NPs are nanoparticles that are synthesized using environmentally friendly methods, such as using plant extracts or other natural sources. Green synthesis of NPs involves the use of environmentally friendly methods to produce NPs without the use of hazardous chemicals. This approach has gained increasing attention in recent years due to its potential for producing biocompatible and non-toxic materials that can be used in a wide range of applications, including photocatalysis [42].

Green synthesis of NPs for photocatalytic applications typically involves the use of plant extracts, microorganisms, or other natural sources as reducing and stabilizing agents [43]. These methods offer several advantages over traditional synthesis methods, such as the use of toxic chemicals, high temperatures, and high pressures. Green synthesis methods are also relatively simple and cost-effective, making them ideal for large-scale production. This method also offers several advantages over other green synthesis methods, such as microorganism-mediated synthesis or physical methods. Plant-mediated synthesis of NPs has been reported to be effective for the synthesis of a wide range of metallic NPs, including silver [44], gold [45], platinum [46], and copper [47]. For instance, nanoparticles synthesized using plant extracts can have a higher degree of crystallinity, smaller particle size, and higher surface area compared to those synthesized using traditional methods [48]. These properties can enhance the photocatalytic activity of the NPs, leading to more efficient degradation of food and juice toxins. Green synthesis of NPs using plant extract mainly include collection of the plant material (root, stem, or leaves) followed by air drying, which usually takes 5–6 days. Then the dried parts are thoroughly grinded, and the weighed quantity is boiled in water, usually at 70–80 °C, for 3–4 h. After filtration, the plant extract is obtained. A salt solution and plant extract are allowed to mix together, which usually results in the synthesis of NPs, either by the precipitation method or the combustion method. The various phytochemicals present in the plant extract serve as reducing, stabilizing and capping agents [49].

Characterization of NPs is an essential step in evaluating their photocatalytic properties. Common techniques used for nanoparticle characterization include UV-Visible spectroscopy, X-ray diffraction (XRD), scanning electron microscopy (SEM), transmission electron microscopy (TEM), and Fourier transform infrared spectroscopy (FTIR) and XPS.

Green-synthesized NPs can provide insights into the optimal photocatalyst properties for effective toxin degradation. The use of green synthesis methods can also enhance the sustainability and eco-friendliness of the overall process [50]. Therefore, studying the green synthesis and characterization of NPs for this application is crucial for the development of efficient and sustainable photocatalytic degradation methods for food and juice toxins.

**Figure 3:**
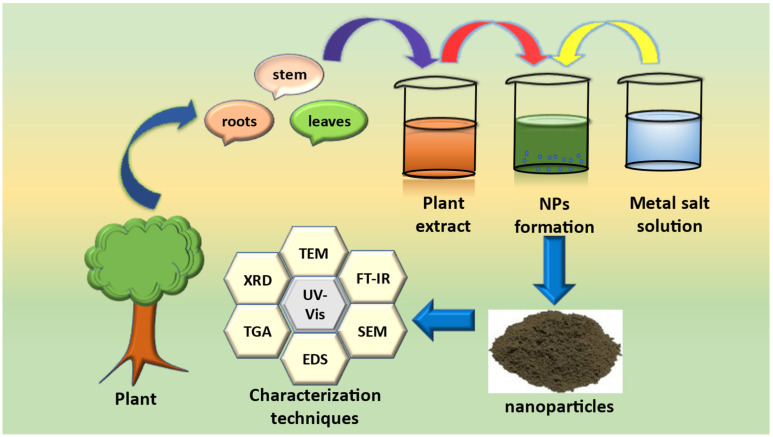
Schematic representation for green synthesis of nanoparticles.

For example, spherical-shaped silver NPs were successfully synthesized using *Viburnum opulus* fruit extract [51]. Another group of researchers also synthesized spherical-shaped silver NPs using the leaf extract of *Kalanchoe brasiliensis* [44]. A schematic representation for the green synthesis of nanoparticles is shown in Figure 3.

## 3. Mechanism of Dye Degradation by Metal and Metal Oxide NPs

The process of photodegradation involves the use of light to break down the chemical bonds in the dye molecules, resulting in the degradation of the dye into smaller, less harmful compounds [52,53]. Green-synthesized NPs can enhance the photodegradation process by acting as a catalyst, absorbing the light and generating reactive oxygen species that can react with the dye molecules and accelerate their degradation [54]. The mechanism of dye degradation by metal NPs involves a series of steps that ultimately lead to the degradation of the dye molecule. This mechanism can be applied to the degradation of food and juice toxins as well.

The first step in the mechanism is the absorption of light by the metal NPs, which promotes the excitation of electrons to higher energy levels. This results in the formation of electron-hole pairs on the surface of the NPs. The excited electrons and holes can then participate in various redox reactions with the dye molecules or food and juice toxins. For example, the excited electrons can reduce the dye molecules, while the holes can oxidize them or react with water molecules to form hydroxyl radicals (^•^OH), a highly reactive species that can degrade the dye molecules or food and juice toxins. In addition, the metal nanoparticles can catalyze the reduction of dissolved oxygen in the solution to form superoxide radicals (O_2_^•−^) or hydrogen peroxide (H_2_O_2_), which can also contribute to the degradation of the dye molecules or food and juice toxins [55].

**Scheme 1:**
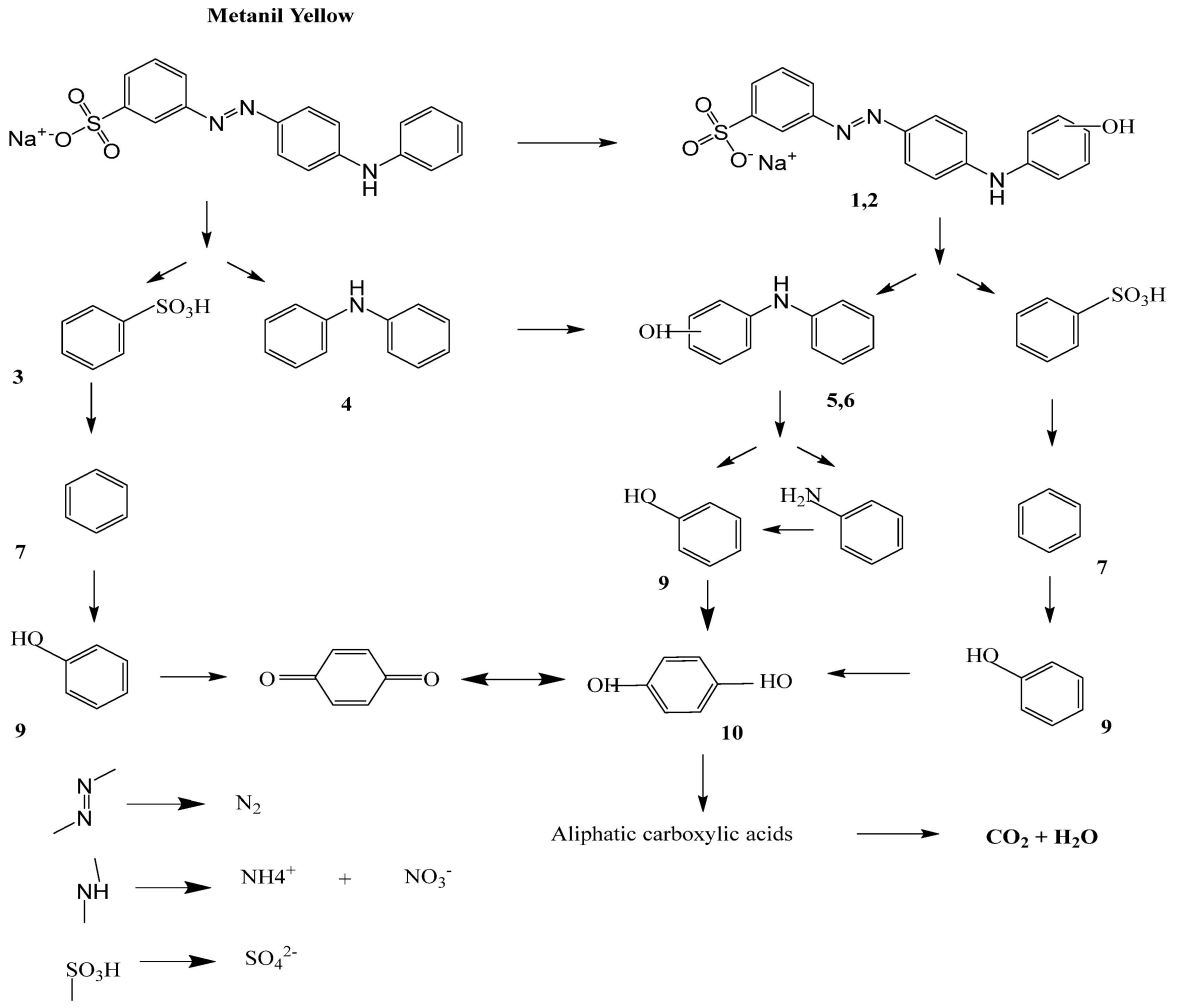
Proposed mechanism for photocatalytic degradation of metanil yellow dye using TiO_2_ NPs [56].

A proposed mechanism for the degradation of metanil yellow using TiO_2_ has been proposed in the literature [56], which is shown in Scheme 1.

In this mechanism, cleavage of the very reactive azo group bond takes place and results in benzene sulphonic acid (3) and diphenyl amine (4) while (1,2) represents two primary hydroxylated products that are identified as isomer intermediates (5,6). This represents benzene sulphonic acid and two hydroxyl-diphenyl amine isomers that are obtained as a result of azo bond cleavage of (1,2). Benzene sulphonic acid undergoes desulphonation to produce benzene while the cleavage of the NH-bond in (5,6) results in benzene (7), aniline (8), and phenol (9). Phenol is then attacked by OH^•^ to form hydroquinone (10), which can lead to the opening of the aromatic ring, providing low-molecular-weight aliphatic carboxylic acids that are ultimately oxidized to carbon dioxide and water.

**Scheme 2:**
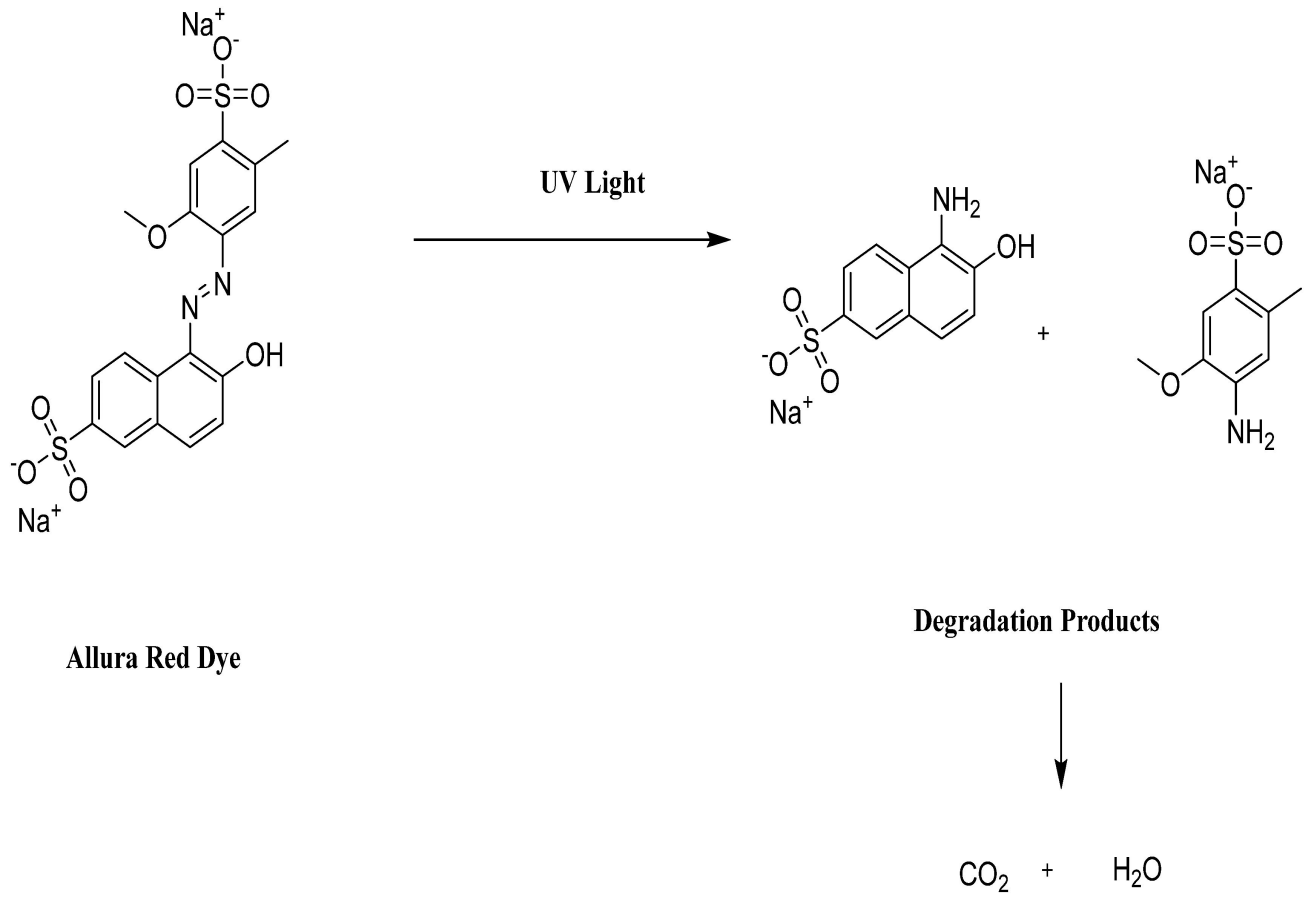
Proposed mechanism for photocatalytic degradation of Allura red AC using CuO NPs [36].

Similarly, photodegradation of Allura red AC dye to create water and carbon dioxide with CuO NPs while giving degradation products as intermediates is shown in Scheme 2 [36].

**Scheme 3:**
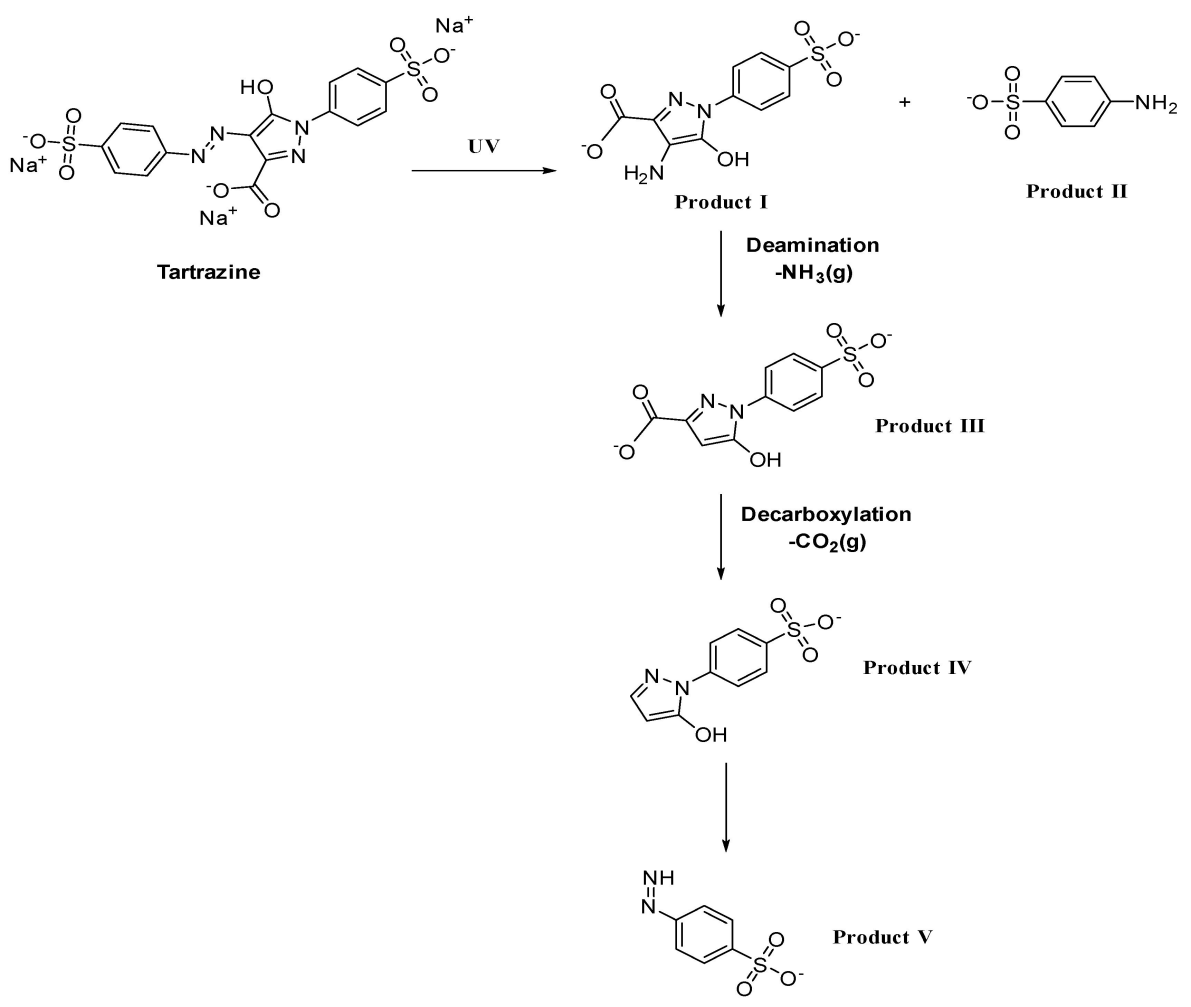
Scheme for the photocatalytic degradation of tartrazine dye under UV irradiation [57].

The degradation mechanism for tartrazine has been reported by a group of researchers and is shown in Scheme 3, and all proposed intermediates have been confirmed using mass spectrometric analysis. In this mechanism for tartrazine, upon irradiation with the UV light, cleavage of (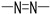) takes place, which results in the formation of subsequent degradation products, i.e., product I and product II. Deamination in product I results in formation of product III, followed by decarboxylation, resulting in product IV. Finally, ring opening takes place with the removal of acrylaldehyde, forming product V [57].

**Figure 4:**
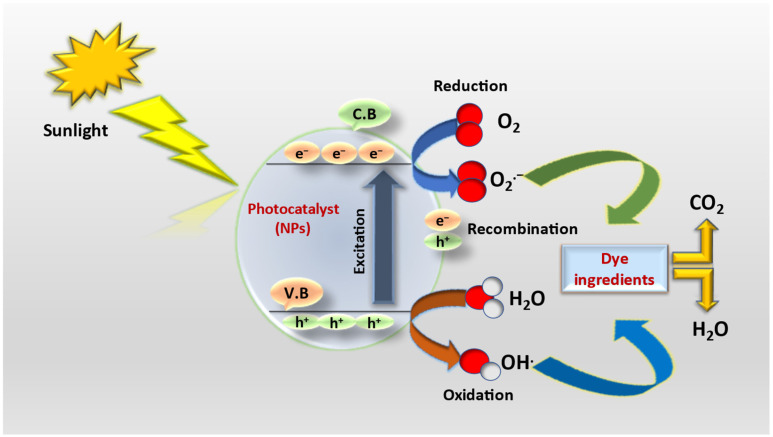
Schematic representation for mechanism of photocatalytic dye degradation.

A general schematic representation for photocatalytic degradation of food toxins (preferably dyes) is shown in Figure 4.

### 3.1. Factors Influencing Degradation of the Dyes

The mechanism of dye or toxin degradation by metal and metal oxide NPs is influenced by several factors, including the size, shape, concentration, and surface properties of the NPs, the type of dye or toxin being degraded, and the pH and temperature of the reaction system [58]. The choice of photocatalyst can affect the efficiency of the degradation process. Different types of photocatalysts have different band gaps, electronic structures, and surface properties, which can affect their ability to generate reactive species and degrade the target compounds. The size, shape, and surface properties of metallic NPs can be tuned to optimize their photocatalytic performance [59]. For example, smaller NPs tend to have higher surface areas, leading to higher photocatalytic activity [60].

The concentration of the photocatalyst is another important factor in determining the rate of photodegradation. For example, in a research study that was carried out for the photocatalytic degradation of methylene blue using green-synthesized CuO NPs, the catalyst concentration was optimized between 0.01 g and 0.07 g, with a constant dye concentration of 0.06 g being the optimized concentration corresponding to maximum degradation of the dye, which was equal to about 86% within 60 min [61]. Similarly, in another experiment, during the photocatalytic degradation of EBT with ZnO NPs, the concentration of the nanoparticles was changed from 10–30 mg/50 mL, and it was observed that, initially, there was an increase in catalytic efficiency up to 99.83%, with 20 mg due to increase in the number of active sites being exposed to light. This was followed by a decline in the photocatalytic efficiency to 84.57% with a further increase in catalyst dose. Therefore, 20 mg was selected as the optimized concentration [62].

The chemical structure and properties of the dye or toxin being degraded can affect the degradation process. Some dyes or toxins may be more resistant to degradation than others, depending on their molecular structure and reactivity. The concentration of the dye or toxin can affect the degradation efficiency. Higher concentrations of the target compound can lead to saturation of the active sites on the photocatalyst surface, leading to decreased degradation efficiency [63].

The pH of the reaction system can also affect the degradation efficiency. Changes in pH can alter the surface charge of the photocatalyst and the target compound, affecting their interaction and the rate of degradation [58,64]. Changes in pH mainly result in the modification of the solid electrolyte interface, ultimately resulting in a direct impact on the sorption–desorption process [56]. It was found that changes in pH from 6.0 to 2.0 increased the degradation efficiency of Congo red dye from 50% to 85% using green-synthesized CuO NPs, and that a decrease in percentage degradation of the dye was observed for an increase in the pH towards basic medium [61].

The intensity of light irradiation can affect the degradation efficiency [65]. Higher light intensities can lead to increased generation of reactive species, leading to faster degradation [58]. The degradation of EBT with ZnO NPs was studied with three different light sources as fluorescent black light, for UV irradiation using wavelength 365-nm, mercury lamp 160 W, and visible light 100 W, and it was found to have a maximum degradation efficiency of 99.24% with fluorescent black light [62].

**Figure 5:**
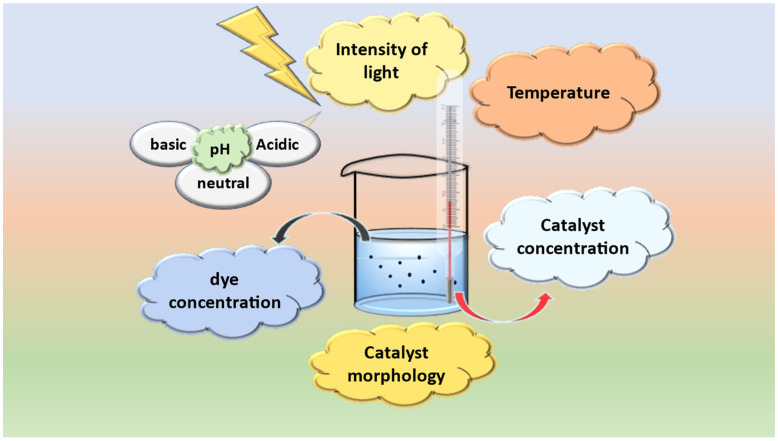
Schematic representation of the different factors influencing rate of photocatalytic degradation.

A schematic representation of different factors influencing the photocatalytic degradation of dyes has been given in Figure 5.

Overall, understanding the factors that influence the degradation of dyes or food and juice toxins during photocatalysis using green photocatalysts is critical for optimizing degradation efficiency and developing effective treatment strategies for these contaminants.

### 3.2. Implementation of Photocatalytic NPs in Dye Degradation

In the context of this topic, metallic NPs are typically used as the active component in green photocatalysts to promote the photocatalytic degradation of toxic compounds. Metallic NPs have unique properties that make them attractive for photocatalysis. These properties include their high surface area, catalytic activity, and the ability to generate reactive oxygen species (ROS) such as hydroxyl radicals under light irradiation. These ROS can effectively degrade food and juice toxins into harmless products.

Photodegradation of tartrazine yellow dye using green-synthesized NPs is a promising approach for the removal of this dye from contaminated water sources [51]. Studies have shown that various types of green-synthesized NPs can be effective in photodegrading tartrazine yellow dye. It has been reported that 76.1% of the tartrazine yellow dye was successfully photodegraded by using ZnO NPs being that were Phyto synthesized using *E. grandis* leaves extract [66]. Another group of researchers successfully synthesized silver NPs using *Aloe vera* plant extract, which were then used for the catalytic degradation of brilliant blue FCF and Tartrazine yellow dyes, respectively, as reported in [67]. The brilliant blue FCF dye was successfully degraded up to 95% in the presence of green-synthesized silver NPs while using NaBH_4_ as a reducing agent. There was also about 55–60% degradation of Tartrazine yellow dye.

In another study, carmine dye was successfully photocatalytically degraded up to 84.08% using silver NPs synthesized using *Kalanchoe brasiliensis* leaves extract [44]. In another study, ZnO and Cu-ZnO NPs were synthesized using leaf extract of Synadium grantii, which had a different percentage composition of doped element. It was found that 3% Cu-ZnO NPs exhibited the best photocatalytic degradation efficiency of 92.2% in 75 min for indigo carmine dye [68]. Another group of researchers reported 98.2% degradation of carmine dye within 140 min using aqueous extract of *F. velutipes* as the biosource [69]. Similarly, 100% degradation of carmine dye has been reported using Cu-Ag bimetallic nanocomposites synthesized using *Citrus paradisi* extract [70].

An updated list of some of the greener synthesized NPs that have been used for the photocatalytic degradation of dyes over the last 3–4 years is given in Table 2.

There are some food dyes that have not yet been degraded by green-synthesized NPs, although there are methods available where chemically synthesized NPs have been used for the photodegradation of those dyes. For example, reports regarding Molybdenum doped cadmium oxide have shown 87.3% degradation of the metanil yellow dye [82]. Reports have also shown 90% degradation of metanil yellow dye by polypyrole-based copper oxide-zinc oxide nanocomposites [83]. On the other hand, ZnO NPs synthesized via a green route using coconut husk were shown to degrade 97% of metanil yellow dye under UV light irradiation [41]. The dye is reported to have 98.7% degradation in the presence of H_2_O_2_ under visible light irradiation [84].

Flowers, such as nickel-cobalt oxide nanosheets, were successfully synthesized via an ecofriendly and cost-effective hydrothermal route which showed an excellent dye-degradation performance of 94.75% towards Allura red AC dye in 10 min under UV light irradiation [85]. There has been 96.12% degradation of the dye in 100 min at a pH of 4 using chitosan-coated nickel selenide nano-photocatalyst; another group of scientists reported that they had achieved 96.99% degradation within 6 min using CuO nanosheets with NaBH_4_ acting as a reducing agent [36] compared to 90.54% degradation using CuO nanorods [86]. Cube-shaped WO_3_ NPs synthesized via a simple hydrothermal method were reported to have 94% degradation of the fast green FCF dye. This specific morphology was been attained using gemini-based twin-tail surfactant as the size and morphology directing agent [87]. In another study about the same dye, 96.1% of the dye was reported to have been degraded within 20 min using salicylic acid sensitized TiO_2_ NPs as compared to 21.3% degradation using only TiO_2_ NPs synthesized via simple sol-gel method [88,89,90,91,92].

Though chemical methods provide excellent methods for the degradation of these dyes, there is still a need to develop green-synthesized NPs for the identified purpose so that pollution can be minimized to a greater extent [93,94,95]. Overall, the use of green-synthesized NPs for photocatalytic degradation of food dyes would offer a sustainable and eco-friendly approach to environmental remediation, with potential applications in wastewater treatment, textile dyeing, and other industries where dye pollution is a concern.

## 4. Advantages and Disadvantages

### 4.1. Advantages

- Environmentally friendly: green synthesis of NPs uses natural materials, such as plant extracts, which are in most cases eco-friendly and non-toxic. Therefore, the use of green-synthesized NPs for photocatalytic degradation of food dyes is an environmentally friendly process.
- Low cost: The green synthesis of NPs is a low-cost process that does not require any expensive equipment or chemicals, making it an affordable process and promising step towards the photocatalytic degradation of food dyes.
- High efficiency: Green-synthesized NPs have been reported to exhibit high photocatalytic activity due to their unique properties, such as high surface area, small particle size, and high reactivity.
- Selectivity: The use of green-synthesized NPs allows for selective degradation of food dyes, as they can be tuned to target specific types of dyes.

### 4.2. Disadvantages

- Lack of standardization: The green synthesis of NPs is still a developing field, and there is a lack of standardization in the methods used to synthesize NPs. This can lead to variability in the properties and performance of the NPs.
- Limited stability: Green-synthesized NPs may have limited stability and can be prone to agglomeration or oxidation, which can affect their performance.
- Toxicity: Although green-synthesized NPs are generally considered to be non-toxic, some plant extracts used for their synthesis may contain toxic compounds. Therefore, it is important to ensure that the NPs are thoroughly characterized and tested for toxicity before use.
- Scale-up challenges: generally, green synthesis of NPs is carried out on smaller scales, so introducing it at industrial levels might be challenging. This can limit the practical application of the NPs for industrial-scale photocatalytic degradation of food dyes.

## 5. Economic Cost

The use of green-synthesized NPs for photocatalytic degradation of various food dyes is an economically friendly process because there is no need to add any expensive chemicals, such as reducing agents or capping agents, during the synthesis, as these compounds are already present in the plant extract.

While introducing green-synthesized NPs at the industrial scale, the economic cost would largely depend on the type of equipment and facilities and on the market demand. High demand would lead to increased production of NPs, resulting in lower costs, but increased production may require more advanced equipment, which can increase the overall costs. Therefore, the economic cost of green-synthesized NPs would vary largely depending upon these factors. A detailed analysis of each factor would be necessary to estimate its economic cost.

## 6. Challenges

Photodegradation of food dyes using green-synthesized NPs is a promising approach for the removal of harmful dyes from food products. However, there are several challenges associated with this method. Some of these include:

- Selection of appropriate NPs: The choice of NPs used for the photodegradation process is critical. The NPs should be stable, non-toxic, and efficient in absorbing light in the visible range. The stability of NPs synthesized using green methods can be a challenge as they may be prone to aggregation, leading to reduced efficiency in dye degradation.

Additionally, the surface of the NPs should be functionalized to enhance their interaction with the dye molecules.

- Scalability: The scalability of the green-synthesis method may be a challenge as it may not be possible to produce the large quantities of NPs needed for industrial applications.
- Photocatalytic activity: The photocatalytic activity of the NPs should be high enough to ensure efficient degradation of the dyes. The photocatalytic activity of nanoparticles can be improved by optimizing their size, shape, and surface area.

## 7. Conclusions

The use of green-synthesized NPs for the photodegradation of food dyes is a promising approach for the degradation of these dyes in contaminated sources to make the sources environmentally friendly. This method is sustainable, eco-friendly, and can lead to the production of NPs with enhanced photocatalytic activity, making it a valuable tool in the development of efficient and sustainable photocatalytic degradation methods. This review article has highlighted the significant progress made in the field, showcasing the efficacy of various green photocatalytic nanomaterials, in the degradation of food and juices dyes. The utilization of these nanoparticles offers several advantages, including their high photocatalytic activity, excellent stability, and low cost. Moreover, the use of green photocatalytic nanomaterials promotes sustainability by reducing reliance on harmful chemicals and minimizing the generation of hazardous waste. Further research is needed to optimize the synthesis and application of these NPs for this purpose. It is important to note that the toxicity of food and juice colorants can vary depending on the dose and frequency of consumption. Additionally, some individuals may be more sensitive to certain colorants than others and may experience adverse effects at lower doses. In general, it is a good idea to read food labels, consume these colorants in moderation and to be aware of any potential allergic reactions or other adverse effects. To reduce the potential toxicity risks associated with dyes, it is important to use dyes that have been tested for safety and to use them in accordance with recommended guidelines and regulations. Consumers can also reduce their exposure to potentially toxic dyes by reading product labels and choosing products that use natural or safer alternatives to synthetic dyes.

## Figures and Tables

**Table 1:** Various food dyes, their uses and associated toxicity.

S. No	Name of the Dye	Synonyms	Chemical Structure	Uses	Toxic Effects
1	Allura Red AC	Red 40	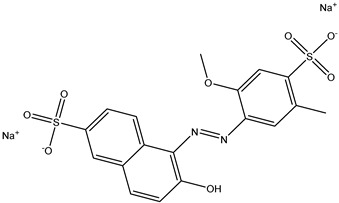	used in candy, baked goods, cereals, and other processed foods [16,17].	causes hyperactivity in children, DNA damage in animal studies, and may be carcinogenic [14,18].
2	Tartrazine	Yellow 5	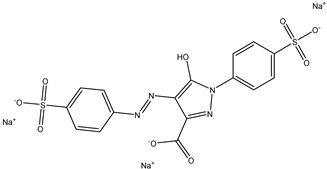	used in processed foods, soft drinks, snacks, and candies [19].	associated with allergic reactions, asthma, hyperactivity in children, kidney tumors in animal studies [20].
3	Brilliant Blue FCF	Blue 1	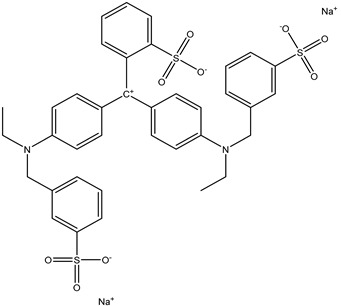	used in sports drinks, candies, baked goods, and other processed foods [21].	causes hyperactivity in children, and it may be carcinogenic [22].
4	Carmine	Red 4	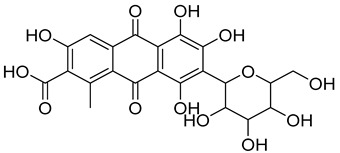	used in many food products, including juices, ice cream, and baked goods [23].	associated with allergic reactions and can cause severe allergic reactions in some individuals [24].
5	Fast green FCF	Green 3	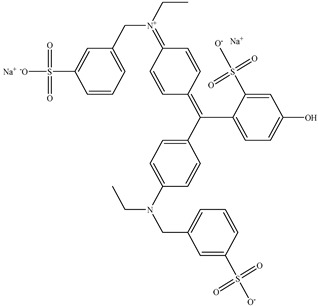	used in processed foods, including candy, ice cream, and baked goods [25].	affects bone marrow chromosomes in animal studies [26].
6	Metanil yellow	Acid yellow 36	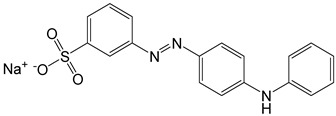	used in a lot of basic food stuffs, sauces, spicy products, ice creams, cold drinks, and juices [21].	mutagenic effects [27], causes damage to gastric mucosa, and often tends to be carcinogenic [28].

**Table 2:** An updated list of various green-synthesized NPs along with their percentage degradation.

S. No	Nanoparticles Synthesized	Plant Source	Dye Type	Degradation Efficiency	Ref.
1	ZnO NPs	*E. grandis* leaves extract	Tartrazine yellow	76.1%	[66]
2	Ag NPs	*Aloe vera* leaf extract	Tartrazine yellow	55–60%	[67]
3	Ag NPs	*Aloe vera* leaf extract	Brilliant blue FCF	95%	[67]
4	Ag NPs	*Kalanchoe brasiliensis* leaves extract	Carmine	84.08%	[44]
5	Ag NPs	*Flammulina velutipes* as biosource	Carmine	98.2%	[69]
6	Cu and Ag bimetallic nanocomposites	*Citrus paradisi* extract	Carmine	100%	[70]
7	ZnO NPs	*Phoenix dactylifera* waste	Methylene blue and eosin yellow dyes	90%	[71]
8	Fe_2_O_3_ NPs	pomegranate seeds extract	Reactive blue	95.08%	[72]
9	Fe_2_O_3_ NPs	*Carica papaya* leaf extract	Remazol yellow RR dye	76.6%	[73]
10	Cu-ZnO NPs	*Synadium grantii* leaf extract	Methylene blue	91.3%	[68]
11	Cu-ZnO NPs	*Synadium grantii* leaf extract	Indigo carmine	92.2%	[68]
12	Cu-ZnO NPs	*Synadium grantii* leaf extract	Rhodamine B	90.1%	[68]
13	ZnO NPs	*Alchemilla vulgaris* (Lady’s mantle) leaves extract	Rhodamine B	75%	[74]
14	Fe_2_O_3_ NPs	*Artocarpus heterophyllus* peel extract	Fuchsin Basic dye	87.5%	[75]
15	Fe_2_O_3_ NPs	*Teucrium polium* leaf extract	Methyl orange	73.6%	[76]
16	Ni@Fe_3_O_4_ and CuO NPs	*Euphorbia maculata* extract	Congo red	88.8%	[61]
17	NiO NPs	*Senna auriculata* aqueous flower extract	Methylene blue	90%	[77]
18	TiO_2_ NPs	mulberry plant extract	Methylene blue	96%	[78]
19	Ag and Au NPs	*Sargassum ilicifolium*	Malachite green	82.9%	[79]
20	MgO NPs	*Camellia sinensis* extract	Methylene blue	97%	[80]
21	ZnO NPs	*Areca catechu* extract	Nigrosin	96%	[81]
22	ZnO NPs	*Areca catechu* extract	Rhodamine B	97%	[81]

## Data Availability

The data presented in this study are available on request from the corresponding author.
